# *APOE* ε4 is associated with younger age at ischemic stroke onset but not with stroke outcome

**DOI:** 10.1212/WNL.0000000000008459

**Published:** 2019-11-05

**Authors:** Cecilia Lagging, Erik Lorentzen, Tara M. Stanne, Annie Pedersen, Martin Söderholm, John W. Cole, Katarina Jood, Robin Lemmens, Chia-Ling Phuah, Natalia S. Rost, Vincent Thijs, Daniel Woo, Jane M. Maguire, Arne Lindgren, Christina Jern, on behalf of the Genetics of Ischaemic Stroke Functional Outcome (GISCOME) network and the International Stroke Genetics Consortium

**Affiliations:** From the Department of Laboratory Medicine (C.L., T.M.S., A.P., C.J.), Institute of Biomedicine, the Sahlgrenska Academy, University of Gothenburg, Gothenburg, Sweden; Department of Clinical Genetics and Genomics (C.L., A.P., C.J.), Sahlgrenska University Hospital, Gothenburg, Sweden; Bioinformatics Core Facility (E.L.), University of Gothenburg, Sweden; Department of Clinical Sciences Lund (M.S., A.L.), Neurology, Lund University, Sweden; Department of Neurology and Rehabilitation Medicine (M.S.), Neurology, Skåne University Hospital, Malmö, Sweden; Department of Neurology (J.W.C.), Baltimore VA Medical Center and University of Maryland School of Medicine, Baltimore, MD; Department of Neurology (K.J.), Sahlgrenska University Hospital, Gothenburg, Sweden; Department of Clinical Neuroscience (K.J.), Institute of Neuroscience and Physiology, the Sahlgrenska Academy University of Gothenburg, Sweden; Neurosciences (R.L.), Experimental Neurology, KU Leuven—University of Leuven; VIB—Center for Brain & Disease Research (R.L.); Department of Neurology (R.L.), University Hospitals Leuven, Belgium; Department of Neurology (C.-L.P.), Washington University School of Medicine in St. Louis; J. Philip Kistler Stroke Research Center (N.S.R.), Department of Neurology, Massachusetts General Hospital, Harvard Medical School, Boston; Stroke Division (V.T.), Florey Institute of Neuroscience and Mental Health, University of Melbourne, Heidelberg, Victoria, Australia; Department of Neurology (V.T.), Austin Health, Heidelberg, Victoria, Australia; Department of Neurology and Rehabilitation (D.W.), University of Cincinnati College of Medicine, OH; Faculty of Health (J.M.M.), University of Technology Sydney, Sydney, Australia; Hunter Medical Research Centre (J.M.M.), Newcastle, Australia; and Department of Neurology and Rehabilitation Medicine (A.L.), Neurology, Skåne University Hospital, Lund, Sweden.

Stroke outcome is determined by a complex interplay, where age and stroke severity are predominant predictors. Studies on hemorrhagic stroke indicate that *APOE* genotype is a predictor of poststroke outcomes,^1,2^ but results from studies on ischemic stroke are more conflicting.^1,3^ There is 1 study suggesting an influence of *APOE* genotype on age at ischemic stroke onset,^4^ and sex-specific effects on outcome have been reported.^5^ Taken together, there is a need for larger studies on *APOE* and ischemic stroke outcomes with integrated information on age, severity, and sex.

The 3 common *APOE* alleles ε2, ε3, and ε4 can be separated by a combination of 2 single nucleotide polymorphisms (SNPs), rs429358 and rs7412. Thus, associations with *APOE* alleles are not directly captured in a regular genome-wide association study (GWAS), where each SNP is investigated separately. We derived the 3 common *APOE* alleles and investigated the interplay between *APOE*, age at ischemic stroke onset, severity, sex, and outcome within a large international collaboration, the Genetics of Ischaemic Stroke Functional Outcome (GISCOME) network.

## Methods

The design and results of the first GWAS on ischemic stroke outcome within GISCOME have been reported,^6^ and the present study comprises the 6,165 cases included in this GWAS. Each center individually obtained ethical approval and participant consent. Baseline stroke severity was assessed by the NIH Stroke Scale and 3-month functional outcome by the modified Rankin Scale (mRS). Genotyping was performed with SNP arrays with subsequent imputation to the 1000 Genomes Phase 3 reference panel as described.^6^ In the present study, we investigated effects of *APOE* minor alleles ε4 and ε2 separately in comparison to the most common allele ε3. To this end, ε4 allele count was defined as the continuous imputed minor allele dosage of rs429358(C), excluding samples with minor allele dosage >0.4 for rs7412(T), and vice versa for ε2, as depicted in figure, A. Each cohort was analyzed separately, and for each analysis, cohorts with an effective number of minor alleles ≤5 or an extreme effect size (β > 100) were excluded. Results from the remaining cohorts were meta-analyzed using inverse variance-weighted fixed effects, unless there were signs of heterogeneity (*p*_*heterogeneity*_ ≤ 0.05) in which case random effects were used.

**Figure:**
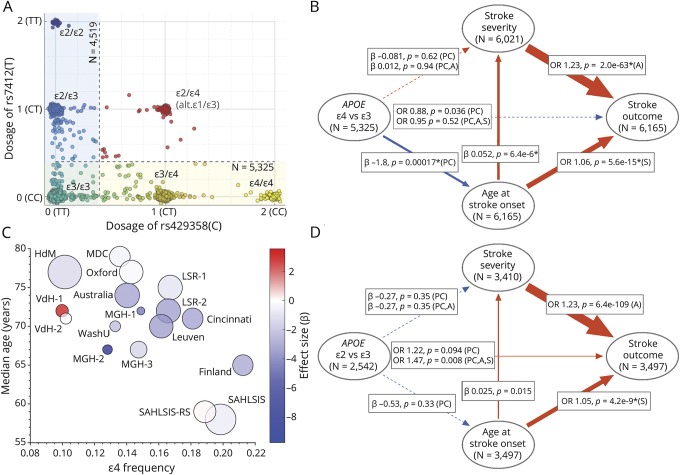
*APOE* allele distribution and associations with age at ischemic stroke onset, stroke severity, and outcome (A) Distribution of *APOE* alleles according to the SNPs rs429358 and rs7412. As the allele counts are inferred from imputation, they are given as a continuum between homozygosity for the major allele and homozygosity for the minor allele, with slightly shifted positions to improve clarity in the graph. In the analyses of ε4 vs ε3, 5,325 cases were included, and 4,519 cases were included in the analyses of ε2 vs ε3. Red positions correspond to cases excluded from both analyses. (B and D) Directed acyclic graphs (DAGs) displaying associations between *APOE* allele count and age at ischemic stroke onset, stroke severity (baseline NIH Stroke Scale score), and dichotomized 3-month mRS score (mRS score 0–2 vs 3–6). N indicates number of cases with nonmissing information, except for *APOE* allele count where N refers to maximum number of cases included in the analysis, that is, cases with allele dosage ≤0.4 for rs7412(T) for ε4 vs ε3 and ≤0.4 for rs429358(C) for ε2 vs ε3. Figure, B examines associations with ε4 allele count and includes both sexes, whereas figure, D displays associations with ε2 allele count in men only. Associations are reported in the squared text boxes as β and *p* value derived from linear regression for associations with age and stroke severity, and OR and *p* value derived from logistic regression for associations with poor outcome (mRS score > 2). Adjustments are indicated in the parentheses as follows: PC, adjusted for ancestry (the 5 first principal components); A, age adjusted; S, stroke severity (baseline NIH Stroke Scale) adjusted. *Refers to result from random effects meta-analysis. Arrow thickness illustrates standardized effect size after the full adjustment specified in the respective text box. Arrow color refers to the direction of the effect. A dotted arrow indicates a nonsignificant association. (C) Bubble chart showing median age at ischemic stroke onset and ε4 allele frequency for individual cohorts in GISCOME. The cohorts are described in Söderholm et al.^6^ Bubble diameter is proportional to the number of cases. Bubble color refers to the effect size (β) of ε4 on age at stroke onset derived from linear regression. GISCOME = Genetics of Ischemic Stroke Functional Outcome; mRS = modified Rankin Scale; OR = odds ratio; SNP = single nucleotide polymorphism.

We used directed acyclic graphs (DAGs) to investigate associations between *APOE*, age at stroke onset, stroke severity, and outcome. A DAG illustrates associations between variables according to a definite direction of causality as depicted by the arrows connecting the variables. For instance, *APOE* can influence age at stroke onset and/or stroke severity, but reverse causality is unlikely as *APOE* genotype is determined at conception. As age and stroke severity are well-established predictors of stroke outcome, we aimed to account for both possible direct effects of *APOE* on outcome and/or indirect effects via associations with age and/or stroke severity as depicted by the 3 different arrows originating from *APOE* in figures, B and D. All genetic analyses were adjusted for ancestry (the 5 first principal components), and adjustments for age and stroke severity were made as indicated (figure, B and D). Prespecified sex-stratified analyses were performed. Associations between allele count, age, and stroke severity were analyzed by linear regression. Associations with outcome were analyzed with logistic (dichotomized mRS score 0–2 vs 3–6) and ordinal logistic regression.

## Results

Increasing allele count of ε4 was associated with younger age at stroke onset (β −1.8, *p* < 0.001, figure, B). This association was consistent across a majority of cohorts (figure, C), significant in both sexes and in cases with first-ever stroke only (data not shown). There was an association between ε4 allele count and favorable outcome (mRS score ≤2) when adjusting only for ancestry, but this association was no longer retained after additional adjustment for age and stoke severity (figure, B).

For ε2 allele count, we found a direct association with poor outcome (mRS score >2) in men after adjustment for ancestry, age, and stroke severity (figure, D). No such association was detected in the whole sample or in women. Neither ε4 nor ε2 allele count showed association with stroke severity.

## Discussion

This is the largest meta-analysis with combined information on common *APOE* alleles, age at ischemic stroke onset, severity, and outcome to our knowledge. We found that increasing ε4 allele count was associated with younger age at stroke onset, which is in line with a previous meta-analysis of candidate gene studies.^4^ However, we found no evidence of a direct effect of ε4 on outcome, similar to 1 recent candidate gene study (N = 786)^7^ and 1 meta-analysis (N = 1,453).^1^

Future studies should elucidate the biological mechanisms behind the association between *APOE* ε4 allele count and younger age at ischemic stroke onset. However, possible mechanisms include effects of altered lipid metabolism. In a pooled analysis, where associations between *APOE* genotype and several biomarkers were investigated, there was an apparent dose-response segregation of low-density lipoprotein cholesterol concentrations by *APOE* genotype, with the highest values in subjects homozygote for the *APOE* ε4 allele.^8^ Furthermore, the same ordering was observed for increasing carotid intima-media thickness and risk of ischemic stroke.^8^

In the sex-stratified analysis, we found an association between increasing ε2 allele count and poor outcome in men. Sex-specific effects of *APOE* on ischemic stroke outcome have been reported^5^ and are not unreasonable to assume from a cardiovascular viewpoint. The ε2 allele has been associated with increasing white matter disease (WMD) in patients with ischemic stroke,^9^ and WMD is in turn associated with poor stroke outcome. Our results might thus be related to a higher prevalence of WMD in male ε2 carriers. However, as we lacked data on WMD for all participants, this hypothesis remains speculative.

The GISCOME study has the advantage of being the largest sample of genetic and ischemic stroke outcome data available. Study limitations have been previously discussed.^6^ In addition, the sample size for the sex-stratified analyses in our present study was small, and we used imputed values from SNP arrays to establish common *APOE* alleles. However, imputation based on the 1000 Genomes reference panel has been reported reliable in inferring these *APOE* alleles.^10^

In conclusion, this study shows that *APOE* ε4 carriers have a younger age at ischemic stroke onset. We also detected worse functional outcome in male ε2 carriers, a result needing replication. Given these findings, even larger studies would be of interest to investigate associations between *APOE* alleles and ischemic stroke outcomes in different age and sex strata.
